# Antidepressant-Like Activity of the Ethanolic Extract from *Uncaria lanosa* Wallich var. *appendiculata* Ridsd in the Forced Swimming Test and in the Tail Suspension Test in Mice

**DOI:** 10.1155/2012/497302

**Published:** 2012-04-09

**Authors:** Lieh-Ching Hsu, Yu-Jen Ko, Hao-Yuan Cheng, Ching-Wen Chang, Yu-Chin Lin, Ying-Hui Cheng, Ming-Tsuen Hsieh, Wen Huang Peng

**Affiliations:** ^1^School of Chinese Pharmaceutical Sciences and Chinese Medicine Resources, College of Pharmacy, China Medical University, No. 91 Hsueh-Shih Road, Taichung 404, Taiwan; ^2^Department of Nursing, Chung Jen College of Nursing, Health Sciences and Management, No. 1-10 Da-Hu, Hu-Bei Village, Da-Lin Township, Chia-Yi 62241, Taiwan; ^3^Department of Biotechnology, TransWorld University, No. 1221, Jen-Nang Road, Chia-Tong Li, Douliou, Yunlin 64063, Taiwan

## Abstract

This study investigated the antidepressant activity of ethanolic extract of *U. lanosa* Wallich var. *appendiculata* Ridsd (UL_EtOH_) for two-weeks administrations by using FST and TST on mice. In order to understand the probable mechanism of antidepressant-like activity of UL_EtOH_ in FST and TST, the researchers measured the levels of monoamines and monoamine oxidase activities in mice brain, and combined the antidepressant drugs (fluoxetine, imipramine, maprotiline, clorgyline, bupropion and ketanserin). Lastly, the researchers analyzed the content of RHY in the UL_EtOH_. The results showed that UL_EtOH_ exhibited antidepressant-like activity in FST and TST in mice. UL_EtOH_ increased the levels of 5-HT and 5-HIAA in cortex, striatum, hippocampus, and hypothalamus, the levels of NE and MHPG in cortex and hippocampus, the level of NE in striatum, and the level of DOPAC in striatum. Two-week injection of IMI, CLO, FLU and KET enhanced the antidepressant-like activity of UL_EtOH_. UL_EtOH_ inhibited the activity of MAO-A. The amount of RHY in UL_EtOH_ was 17.12 mg/g extract. Our findings support the view that UL_EtOH_ exerts antidepressant-like activity. The antidepressant-like mechanism of UL_EtOH_ may be related to the increase in monoamines levels in the hippocampus, cortex, striatum, and hypothalamus of mice.

## 1. Introduction

Depression, a widespread incapacitating psychiatric ailment, imposes a substantial health burden on society [1]. Affective disorder are characterized by a disturbance of mood associated with alteration in behavior, energy, appetite, sleep, and weight [2]. According to the most accepted hypothesis of depression, the monoamine theory, patients with major depression have symptoms that are reflected changes in brain monoamine neurotransmitters, specifically norepinephrine (NE) and serotonin (5-HT) [3]. Clinical data suggests that dopamine (DA) is also involved in the pathophysiology and treatment of depression [4]. Medications such as tricyclic antidepressants (TCAs), selective serotonin reuptake inhibitors (SSRIs), monoamine oxidase inhibitors (MAOIs), specific serotonin-norepinephrine reuptake inhibitors (SNRIs), 5-HT_2_ receptor antagonists, and other heterocyclics are clinically employed for drug therapy [5]. However, these drugs can impose a variety of side-effects including sedation, apathy, fatigue, sleep disturbance, cognitive impairment, and sexual dysfunction, and so forth. Hence, there remains a pressing need for new effective and better-tolerated antidepressants. 

Herbal therapies may be effective alternatives in the treatment of depression, such as* Hypericum perforatum L.* [6],* Cordyceps sinensis* [7], and *Perilla frutescens* [8]. The *Uncaria* species recorded in Chinese Pharmacopoeia and Taiwan Herbal Pharmacopoeia include* Uncaria rhynchophylla* (Miquel) Jacks (abbrev. as *UR*), *U. macrophylla *Wallich. *U. hirsuta* Haviland (*UH*), *U. sinensis* (Oliver) Havil, and *U. sessilifructus* Roxburgh [9, 10]. According to Flora of Taiwan, there are three different species of *Gouteng* in Taiwan: *UR*, *UH,* and *U. lanosa* Wallich var*. appendiculata* Ridsd (*UL*) [11]. However, *UL* is not recorded in Pharmacopoeia. In traditional Chinese medicine, *Gouteng* is categorized as a herb to extinguish wind, arrest convulsions, clear heat, and pacify the liver [12]. *Gouteng* is mainly used to treat cardiovascular and central nervous system ailments, including light headedness, convulsions, numbness, and hypertension [12]. Several studies demonstrate that the herb extract mainly acts on neuroprotective effect used to treat antiepileptic [13–15], anti-Parkinsonian [16], anti-Alzheimer's disease [17, 18], anxiolytic [19], protective action against ischemia-induced neuronal damage [20, 21], anti-inflammation [22]. Alkaloids are the active pharmacological component in *Gouteng* and comprise components include RHY, isorhynchophylline, hirsutine, hirsuteine, corynantheine, isocorynoxeine. RHY exhibited a similar pharmacological activity when compared with *Gouteng* [12]. RHY is an important active component of alkaloids separated from gambir plant (*Gouteng* in Chinese), RHY exerts the protective action primarily by inhibiting of NMDA and 5-HT_2_ receptor-mediated neurotoxicity during ischemia [21]. RHY also affects the levels of serotonin in cortex, striatum, hippocampus, and hypothalamus [23, 24]. From the above perspectives, we inferred that RHY is the key component of antidepressant-like activity of *Gouteng*. *Gouteng* possesses neuroprotective effect, regulation of monoamine transporters, macrophage theory [25], and regulation of glutamatergic system [26]. Our preliminary test indicated that ethanolic extract of *U. lanosa* Wallich var*. appendiculata* Ridsd. (UL_EtOH_) contained the largest amount of RHY among *Uncaria* species in Taiwan. However, the antidepressant-like activity of UL_EtOH_ has not been investigated, which encouraged us to investigate the effects of UL_EtOH_ on depression problems.

In the present study, we aimed to investigate the effect of UL_EtOH_ in FST and TST in mice. The behavioral despair tasks have good predictive value for antidepressant potency in humans [27]. Moreover, we investigated whether the effect of UL_EtOH_ in FST and TST is dependent on its interaction with the 5-HT, NE, and DA receptors, and the brain monoamine neurotransmitter concentration. MAO activity was also tested by neurochemical and biochemical assays to confirm the participation of monoamine transmitters in treatment involving UL_EtOH_.

## 2. Materials and Methods

### 2.1. Animals

Male ICR albino mice (weighing around 22 g), purchased from BioLASCO Taiwan Co., Ltd., were used in the present study. They were maintained at 22 ± 1°C with free access to water and food, under a 12 : 12 h light/dark cycle (lights on at 08:00 h). All manipulations were carried out between 9:00 and 15:00 h, with each animal used only once. All procedures in this study were performed in accordance with the NIH Guide for the Care and Use of Laboratory Animals. The experimental protocol was approved by the Committee on Animal Research, China Medical University. The minimum number of animals and duration of observations required to obtain consistent data were used.

### 2.2. Plant Materials

*Uncaria rhynchophylla* (Miquel) Jacks (UR) was collected from SiaoWulai, *U. hirsuta* Haviland (UH) was collected from Wulai, and *U. lanosa* Wallich var*. appendiculata* Ridsd (UL) was collected from Xuhai, Mudan Township of Taiwan, and was identified by Dr. Chao-Lin Kuo, Leader of the School of Chinese Pharmaceutical Sciences and Chinese Medicine Resources (CPCR). The voucher specimen (Number: CMU-CPCR-UL-10001) was deposited at CPCR.

### 2.3. Preparation of Plant Extract

Dried 1 kg of UR, UH, and UL, made from the stems and hooks of plants, were sliced into small pieces and ground into a powder, and extracted four times with 5% ammonia solution and 70% ethanol. The extracts were filtered, combined, and concentrated under reduced pressure at 40°C to obtain the UR_EtOH_, UH_EtOH_, UL_EtOH_ extracts. The yield ratios of the UR_EtOH_, UH_EtOH_, UL_EtOH_ extracts (120 g, 97 g, 115 g) were 12%, 9.7%, 11.5%.

### 2.4. Drugs and Drug Administration

Imipramine HCl (IMI), clorgyline HCl (CLO), maprotiline HCl (MAP), fluoxetine HCl(FLU), bupropion HCl (BUP), ketanserin (KET), sodium octyl sulfate, norepinephrine HCl (NE), dopamine HCl (DA), 5-hydroxytryptamine HCl (5-HT), 4-Hydroxy-3-methoxyphenylglycol (MHPG), 4-dihydroxyphenylacetic acid (DOPAC), and 5-hydroxyindoleacetic acid (5-HIAA), as well as horseradish peroxidase (HRP), benzylamine, amplex red, and phosphate-buffered saline (PBS) solution were purchased from Sigma-Aldorich (St. Louis, MO, USA). RHY was purchased from Matsuura Yakugyo Co., Ltd (Japan). Drugs were dissolved in normal saline, except HRP, benzylamine, and amplex red that was diluted in PBS solution. Citric acid, tri-natriumcitrate-2-hydrate, and EDTA were purchased from Merck. UR_EtOH_, UH_EtOH_, UL_EtOH_, or saline was administered by oral route, whereas the other drugs were administered by i.p. route. The i.p. or p.o. administrations were given in a volume of 10 mL/kg body weight. Tests were performed 1 hr (UL_EtOH_) and 30 min (imipramine, fluoxetine, clorgyline, maprotiline, bupropion, ketanserin) after administration.

### 2.5. HPLC Analysis of UR_EtOH_, UH_EtOH_, and UL_EtOH_

The HPLC system consisted of a Shimadzu (Kyoto, Japan) LC-10ATvp liquid chromatograph equipped with a DGU-14A degasser, an FCV-10ALvp low-pressure gradient flow control valve, an SIL-10ADvp autoinjector, an SPD-M10Avp diode array detector, and an SCL-10Avp system controller. Peak areas were calculated using Shimadzu Class-LC10 software (Version 6.12 sp5). The column was a Phenomenex Synergi 4_ Fusion-RP 80A column (250 mm × 4.6 mm). The gradient mobile phase was methanol (solvent A) and 0.01 mol/L triethylamine, and adjusted to adjust pH to 7.5 with glacial acetic acid (solvent B) solvent A : B = 60 : 40. The sample was injected of 10 *μ*L. The following gradient profile was run at 1.0 mL/min over 60 min. Peaks were detected at 274 nm with SPD-M10AVP (Shimadzu) detector. The peaks of UR_EtOH_, UH_EtOH_, and UL_EtOH_ samples were identified by comparison with the standard solutions (RHY). The UR_EtOH_, UH_EtOH_, and UL_EtOH_ solutions were quantified by spiking with a known amount of standard and also by comparing the area under curve. The repeatability of the method was evaluated by injecting the solution of UR_EtOH_, UH_EtOH_, and UL_EtOH_ and standard solution three times, and the relative standard deviation (RSD) percentage was calculated.

### 2.6. Behavior Despair Study

For FST and TST, animals were divided into six groups (*n* = 10/group): control (0.9% saline), the four doses of UL_EtOH_ (0.0625, 0.125, 0.25, 0.5 g/kg) and 10 mg/kg IMI for 14-day treatment.

#### 2.6.1. Forced Swimming Test (FST)

The method was carried out on mice according to the method of Porsolt et al. [28]. Mice were placed in an open cylindrical container (diameter 10 cm, height 25 cm), containing 15 cm of water at 25 ± 1°C. The duration of observed immobility was recorded during the last 4 min of the 6-minute testing period [29, 30]. Mice are forced to swim in a restricted space from which they cannot escape and are induced to a characteristic behavior of immobility. Each mouse was judged to be immobile when it ceased struggling and remained floating motionless in the water, making only those movements necessary to keep its head above water. Decrease in the duration of immobility during the FST was taken as a measure of antidepressant activity.

#### 2.6.2. Tail Suspension Test (TST)

The total duration of immobility induced by tail suspension was measured according to the method of Steru et al. [31]. Mice both acoustically and visually isolated were suspended 50 cm above the floor by adhesive tape placed approximately 1 cm from the tip of the tail. The time during which mice remained immobile was quantified during a test period of 6 min. Mice were considered immobile only when they hung passively and completely motionless.

### 2.7. Open-Field Test

For open-field test. Animals were divided into five groups (*n* = 6/group): control (0.9% saline), the three doses of UL_EtOH_ (0.125, 0.25, 0.5 g/kg), and 10 mg/kg IMI for 14-day treatment.

To assess the effects of UL_EtOH,_ on locomotor activity, mice were evaluated in the open-field paradigm as previously described [32]. Animals were individually placed in a box (40 × 60 × 50 cm). The mice were placed in the center and their behavior was noted immediately and continued for 5 min. The parameters such as resting time, total movement distance, total movement time, total movement were recorded by video camera and registered in the computer. During the interval of the test the apparatus was cleaned.

### 2.8. Pharmacological Treatments

We investigated whether the antidepressant-like activity of UL_EtOH_ in FST and TST is dependent on its interaction with IMI (a tricyclic antidepressant), MAP (a selective NE reuptake inhibitor), FLU (a selective 5-HT reuptake inhibitors), BUP (a selective DA reuptake inhibitor), CLO (a selective MAO-A inhibitor), and KET (a preferential 5-HT_2A_ receptor antagonist). To this end, mice were pretreated with UL_EtOH_ (0.5 g/kg for two weeks' administration) or saline. They received IMI, FLU, KET, CLO (5 mg/kg for two weeks' administration), MAP (20 mg/kg for two weeks' administration), or BUP (4 mg/kg for two weeks' administration) 30 mins before being tested in FST and TST.

The doses of the drugs which do not affect locomotor activity and immobility time were selected on the basis of literature data [33–35] and our preliminary test.

### 2.9. Determination of Monoamines and Their Metabolites Levels in the Mice Frontal Cortex, Striatum, Hippocampus, and Hypothalamus

Animals were divided into six groups (*n* = 6/group): control (0.9% saline), control versus FST, the three experiment groups (0.125, 0.25, 0.5 g/kg, for two weeks' administration)_,_ and IMI (10 mg/kg for two weeks' administration).

Monoamines were measured according to the method of Renard et al. [36]. Briefly, mice were killed by cervical dislocation without anesthesia just after the FST. The brain was removed after a rapid dissection of frontal cortex, striatum, hippocampus, and hypothalamus were isolated. The four brain tissues were weighed and placed separately in 5 mL of ice-cold homogenizing solution (8.8 mg of ascorbic acid and 122 mg of EDTA in 1000 mL of perchloric acid 0.1 M). After homogenization, the solution was centrifuged at 10,000 ×g for 10 min at 4°C. Twenty microliters of the resultant supernatant was injected in the high-performance liquid chromatography (HPLC) system. The levels of monoamines (NE, DA and 5-HT) and their metabolites (MHPG, DOPAC, 5-HIAA) were measured by HPLC (Waters 610) with electrochemical detection in the three brain tissues. The mobile phase [4.2 g/L] citric acid monohydrate, 6.8 g/L sodium acetate trihydrate, 0.8 g/L octanesulfonic acid sodium salt, 0.05 g/L tetrasodium ethylenediamine tetraacetate, 0.02% (v/v) dibutyl amine, and 7% (v/v) methyl alcohol) was delivered at 1.0 mL/min. The reverse-phase column used was a Merk Lichrospher 100 RP-18 endcapped column with a length of 12.5 cm and an internal diameter of 4.0 mm (E. Merk 50734). The compounds were measured at +0.75 V using a Bioanalytical Systems LC-4C electrochemical detector.

### 2.10. Measurements of Monoamine Oxidase Activity

Animals were divided into five groups (*n* = 6/group): control (0.9% saline), the three doses of UL_EtOH_ (0.125, 0.25, 0.5 g/kg, for two weeks' administration)_,_ and CLO (10 mg/kg for two weeks' administration).

Mice were sacrificed and the brain tissues was rapidly frozen (−80°C) until analyzed. The brain tissues was each homogenized in 50 mM phosphate buffer (pH 7.4) containing 0.5 mM EDTA and 0.25 M sucrose and stored at −80°C. Protein content of the homogenate was determined using the method of Lowry et al. [37]. Mouse brain monoamine oxidase activity was measured following the method of Zhou and Panchuk-Voloshina [38]. Briefly, For the measurement of each type of MAO, serotonin was used as a substrate for MAO-A and benzylamine for MAO-B. The experiments were conducted at room temperature for 60 min in a reaction mixture with brain homogenates at a final protein concentration of 8 mg/mL. For the sensitivity assay, the brain homogenates with different protein concentrations were incubated in a reaction mixture of 200 mM Amplex Red, 1 mM benzylamine, and 1 U/mL HRP at room temperature for 60 min.

### 2.11. Statistical Analysis

All results are expressed as mean ± SEM. Data were analyzed by one-way ANOVA followed by Bonferroni's multiple range test. The criterion for statistical significance was *P* < 0.05. All statistical analyses were carried out by using SPSS for Windows (SPSS Inc.).

## 3. Results

### 3.1. HPLC Analysis of UR_EtOH_, UH_EtOH_, and UL_EtOH_

**Figure 1:**
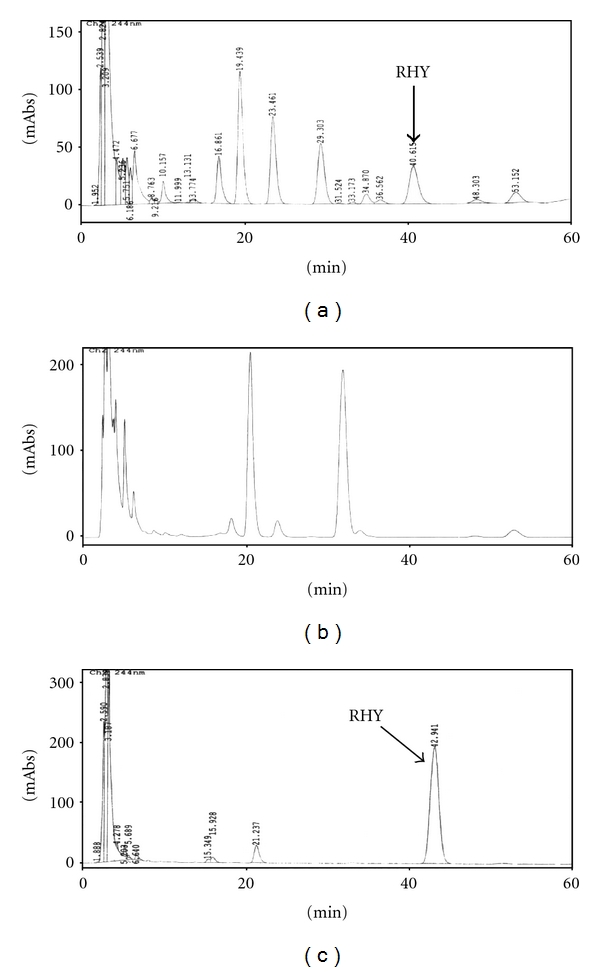
The HPLC chromatographic profiles, at 274 nm, of (a) UR_EtOH_, (b) UH_EtOH_, and (c) UL_EtOH_.

The HPLC chromatogram shows that RHY is the major components among organic molecules of UR_EtOH_, UH_EtOH_, and UL_EtOH_. As shown in Figure 1, the content of RHY in UR_EtOH_ and UL_EtOH_ were 3.87 mg/g and 17.12 mg/g. UH_EtOH_ did not detect the content of RHY. 

### 3.2. Effect of Repeated Treatment with UL_EtOH_ on the Immobility Time Both in the FST and TST

**Figure 2:**
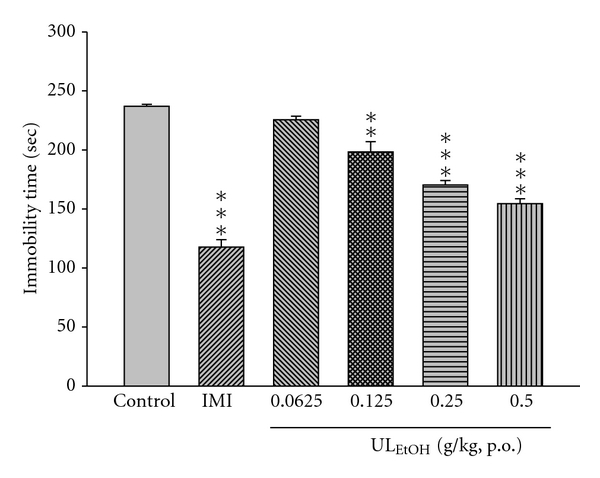
The effect of ethanol extracts from *U. lanosa* (UL_EtOH_, 0.0625–0.5 g/kg, p.o.), or Imipramine (IMI, 10 mg/kg, i.p.) for two weeks' administration on the immobility time in the forced swimming task. The values are mean ± SEM for each group (*n* = 10). **P < 0.01, ***P < 0.001 as compared with control group (one-way ANOVA followed by Bonferroni's multiple range test).

**Figure 3:**
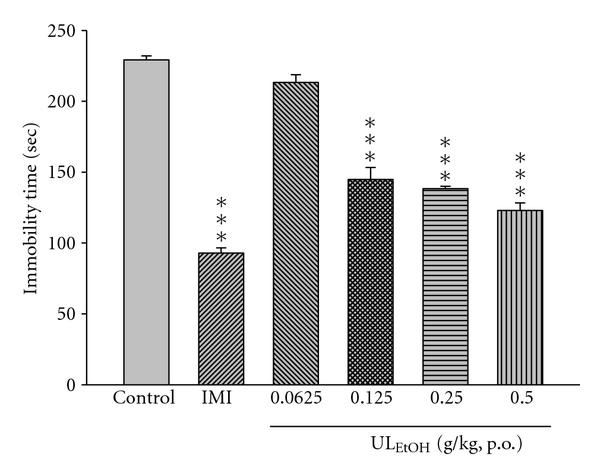
The effect of ethanol extracts from (UL_EtOH_, 0.0625–0.5 g/kg, p.o.), or Imipramine (IMI, 10 mg/kg, i.p.) for two weeks' administration on the immobility time in the tail suspension test. The value are mean ± SEM for each group (*n* = 10). ***P < 0.001 as compared with control group (one-way ANOVA followed by Bonferroni's multiple range test).

In order to investigate whether UL_EtOH_ can produce chronic changes in depression-related behavior in FST and TST, we treated mice with different dosages to mice via continuous oral administration for 14 days. UL_EtOH_ decreased significantly the immobility time in FST (dose range: 0.0625–0.5 g/kg, p.o.; Figure 2). UL_EtOH_ also caused a reduction in the immobility time in TST (dose range: 0.0625–0.5 g/kg, p.o.; Figure 3). In both tests, IMI at doses of 10 mg/kg produced a reduction of the immobility time that was stronger than that afforded by UL_EtOH_ (Figure 3). 

### 3.3. Effect of Repeated Treatment with UL_EtOH_ on the Locomotor Activity in Mice

**Figure 4:**
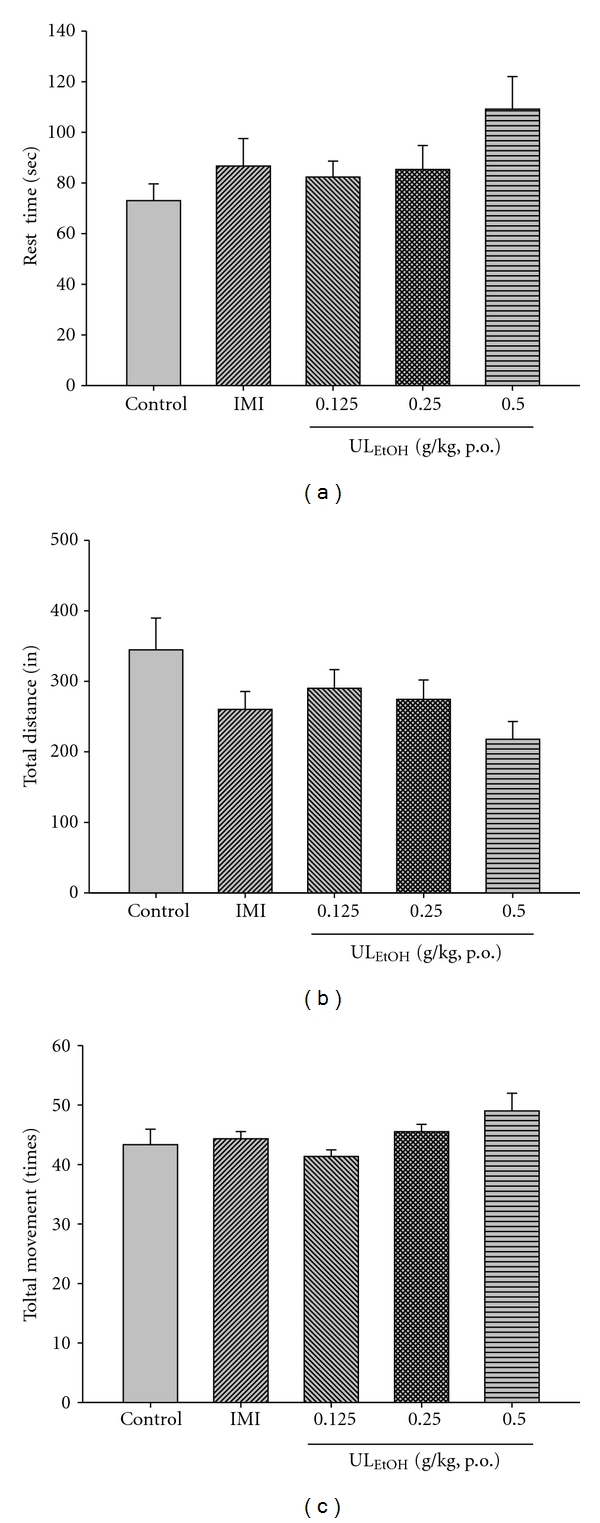
The effects of UL_EtOH_ for two weeks administration on resting time, total movement distance and total movement time in the locomotor. The value are mean ± SEM for each group (*n* = 6).

In order to determine whether UL_EtOH_ actually possesses an antidepressant-like activity, we tested the locomotion counts to exclude the excitatory or inhibitory effects after administration of UL_EtOH_. UL_EtOH_ did not affect locomotor activity at the same doses that significantly reduced immobility response in the FST and TST (Figure 4).

### 3.4. Effect of Combination of UL_EtOH_ with IMI, FLU, CLO, MAP, BUP, and KET on Immobility Periods in FST and TST

**Figure 5:**
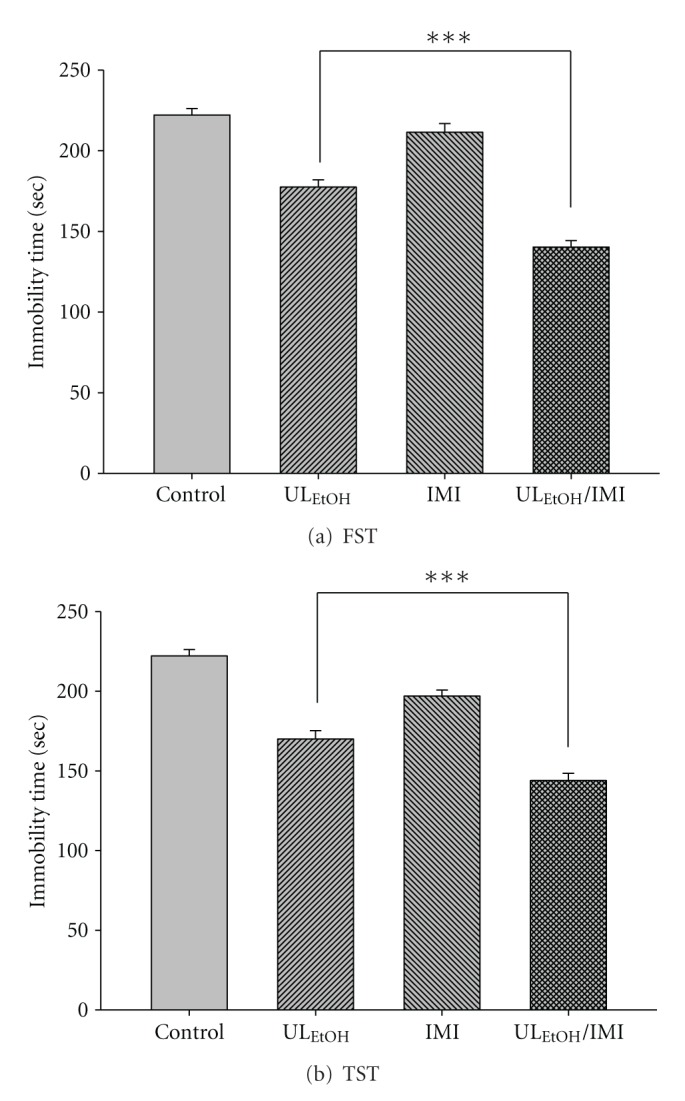
Effect of imipramine(IMI)on UL_EtOH_ -induced immobility time in (a) FST and (b) TST. The value are mean ± SEM for each group (*n* = 6). ***P < 0.001 as compared with UL_EtOH_ alone.

The results depicted in Figure 5 show the effect of treatment of mice with IMI (5 mg/kg for two weeks' administration, a dose that did not affect the immobility time) on the reduction in immobility time elicited by UL_EtOH_ (0.5 g/kg, p.o.). Post-hoc analyses indicated that the treatment of mice with IMI augmented the antidepressant-like activity of UL_EtOH_ in FST and TST.

**Figure 6:**
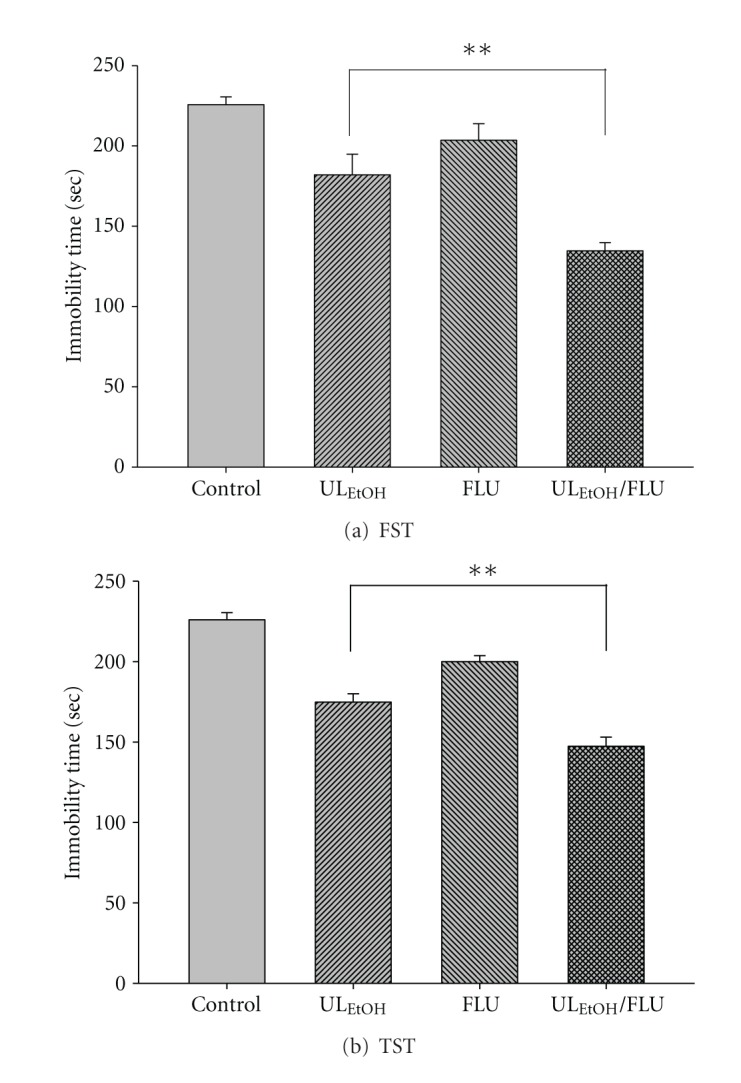
Effect of fluoxetine (FLU)on UL_EtOH_-induced immobility time in (a) FST and (b) TST. The value are mean ± SEM for each group (*n* = 6). **P < 0.01 as compared with UL_EtOH_ alone.

The results depicted in Figure 6 show the effect of treatment of mice with FLU (5 mg/kg for two weeks' administration, a dose that did not affect the immobility time) on the reduction in immobility time elicited by UL_EtOH_ (0.5 g/kg, p.o.). Post-hoc analyses indicated that the treatment of mice with FLU augmented the antidepressant-like activity of UL_EtOH_ in FST and TST.

**Figure 7:**
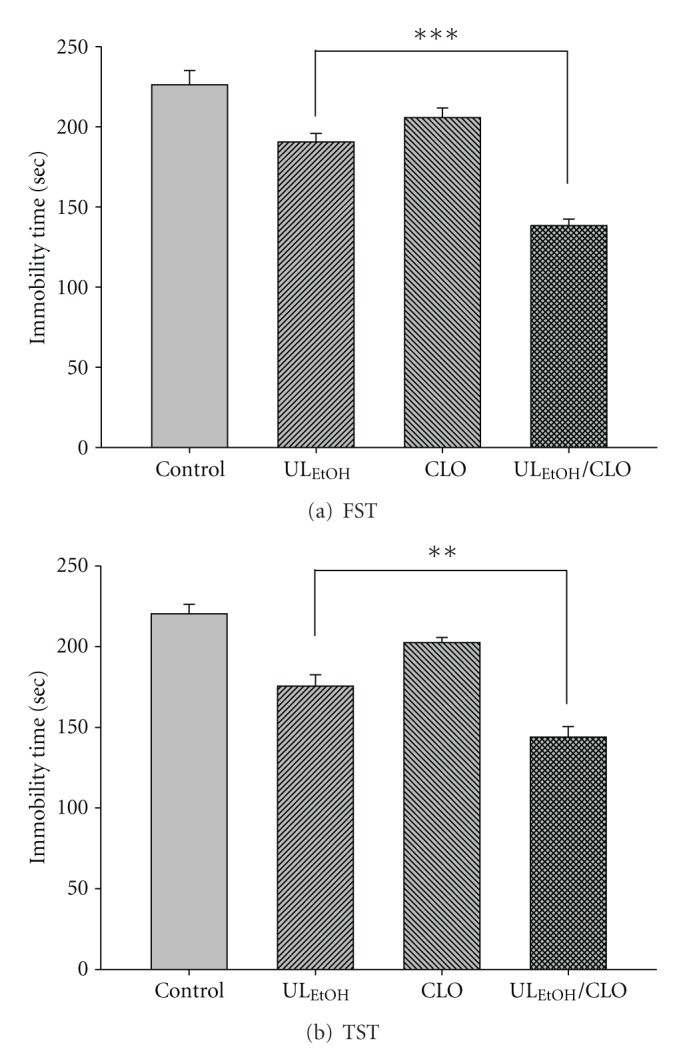
Effect of clorgyline (CLO)on UL_EtOH_-induced immobility time in (a) FST and (b) TST. The value are mean ± SEM for each group (*n* = 6). **P < 0.01***P < 0.001 as compared with UL_EtOH_ alone.

The results depicted in Figure 7 show the effect of treatment of mice with CLO (5 mg/kg for two weeks' administration, a dose that did not affect the immobility time) on the reduction in immobility time elicited by UL_EtOH_ (0.5 g/kg, p.o.). Post-hoc analyses indicated that the treatment of mice with CLO augmented the antidepressant-like activity of UL_EtOH_ in FST and TST.

**Figure 8:**
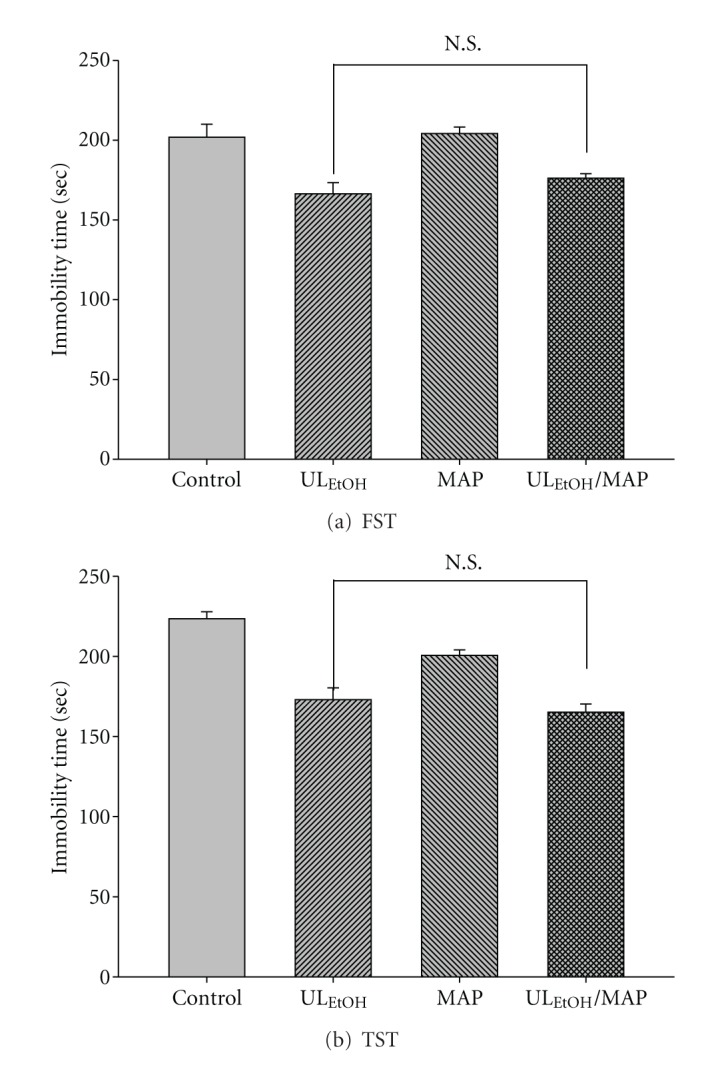
Effect of maprotiline (MAP) on UL_EtOH_-induced immobility time in (a) FST and (b) TST. The value are mean ± SEM for each group (*n* = 6). N.S.: nonsignificant.

The results depicted in Figure 8 show the effect of treatment of mice with MAP (20 mg/kg, for two weeks' administration, a dose that did not affect the immobility time) on the reduction in immobility time elicited by UL_EtOH_ (0.5 g/kg, p.o.). Post-hoc analyses indicated that the treatment of mice with MAP did not augment the antidepressant-like activity of UL_EtOH_ in FST and TST.

**Figure 9:**
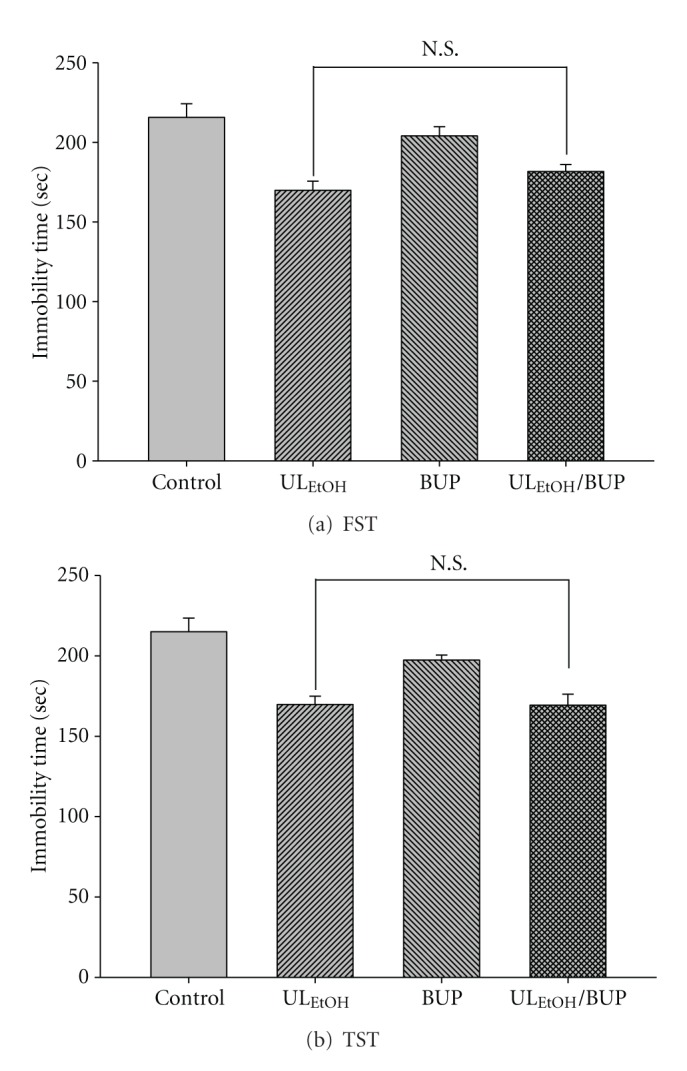
Effect of bupropion (BUP) on UL_EtOH_-induced immobility time in (a) FST and (b) TST. The value are mean ± SEM for each group (*n* = 6). N.S.: nonsignificant.

The results depicted in Figure 9 show the effect of treatment of mice with BUP (4 mg/kg, for two weeks' administration, a dose that did not affect the immobility time) on the reduction in immobility time elicited by UL_EtOH_ (0.5 g/kg, p.o.). Post-hoc analyses indicated that the treatment of mice with BUP did not augment the antidepressant-like activity of UL_EtOH_ in FST and TST.

**Figure 10:**
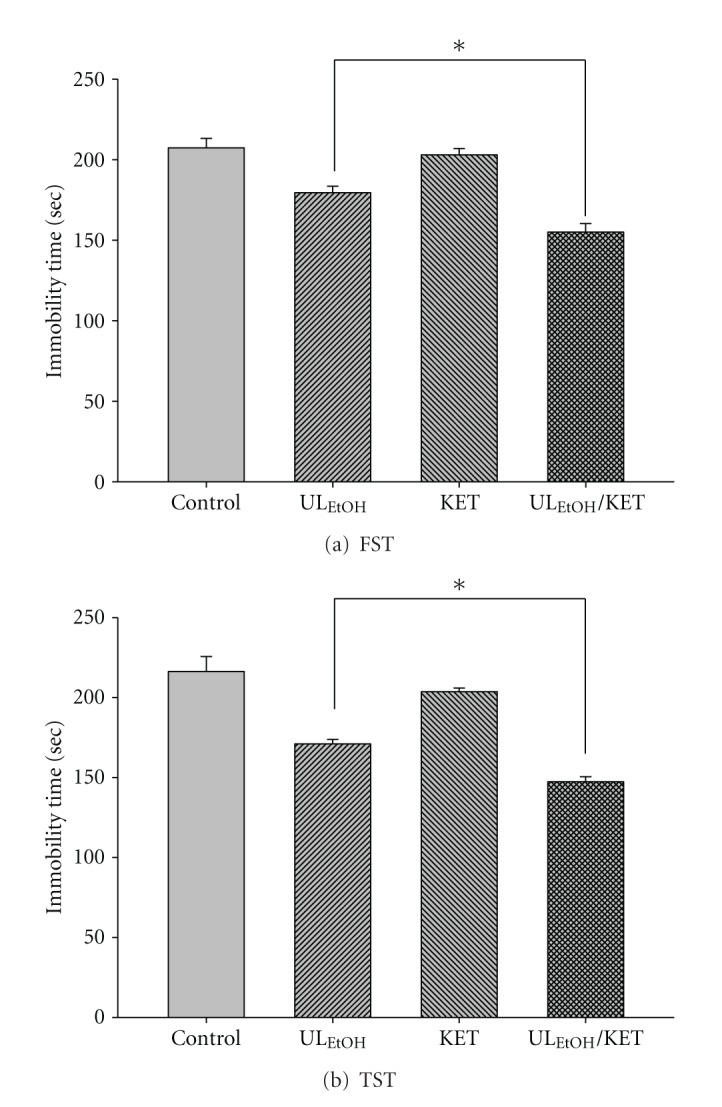
Effect of ketanserin (KET) on UL_EtOH_-induced immobility time in (a) FST and (b) TST. The value are mean ± SEM for each group (*n* = 6). *P < 0.05 as compared with UL_EtOH_ alone.

The results depicted in Figure 10 show the effect of treatment of mice with KET (5 mg/kg for two weeks' administration, a dose that did not affect the immobility time) on the reduction in immobility time elicited by UL_EtOH_ (0.5 g/kg, p.o.). Post-hoc analyses indicated that the treatment of mice with KET augmented the antidepressant-like activity of UL_EtOH_ in FST and TST.

### 3.5. Determination of Monoamines and Their Metabolites Levels in the Mice Frontal Cortex, Striatum, Hippocampus, and Hypothalamus

The concentrations of NE, DA,  5-HT, and its metabolites in the frontal cortex, striatum, hippocampus, and hypothalamus are presented in Tables 1, 2, 3, and 4. UL_EtOH_ (0.125 g/kg, p.o.) increased the level of NE in hypothalamus, and the level of DOPAC in striatum. UL_EtOH_ (0.25 g/kg, p.o.) increased the level of 5-HT in cortex and striatum, the level of 5-HIAA in striatum, hippocampus, and hypothalamus, and the level of NE in cortex, hippocampus, the level of MHPG in hippocampus,and level of DOPAC in striatum. UL_EtOH_ (0.5 g/kg, p.o.) increased the levels of 5-HT and 5-HIAA in cortex, striatum, hippocampus, and hypothalamus, the levels of NE and MHPG in cortex and hippocampus, the level of NE in striatum, and level of DOPAC in striatum.

### 3.6. Measurements of Monoamine Oxidase Activity

Table 5 summarizes the effect of UL_EtOH_ and clorgyline on the activities of type A and type B monoamine oxidase in mouse brain. UL_EtOH_ (0.5 g/kg) and clorgyline (10 mg/kg) inhibited the activity of type A monoamine oxidase in the mouse brain.

## 4. Discussion

In the present study, we analyzed the RHY content of *Gouteng* grown in Taiwan and choose the *UL* which has higher amount of RHY as the research sample. To raise the yield ratio of alkaloid, the researcher alkalized the three species of *Gouteng* by 5% ammonia solution, turning alkaloid salts into free alkaloid, followed by 70% ethanol extracting. After the above procedure, the UR_EtOH_, UH_EtOH_, and UL_EtOH_ were produced. Afterwards, the researcher used HPLC method to analyse the RHY content of the samples. The analytical result of UL_EtOH_ contained most RHY among all. However, from the study of Jung et al. [19], the aqueous extract of *UR* (UR_DDW_) possesses anxiolytic activity by inhibiting WAY1005635 (the compounds that could selective block 5-HT_1A_ presynaptic receptors and prevent the negative feedback might be effective) [34]. In this study, the researcher analysed the RHY content of UR_DDW_. Result showed that UR_DDW_ did not detect RHY (unpublished data) and suggested that the antianxiety activity of UR_DDW_ was not related to RHY. 

The forced swimming and tail suspension tests are behavioral despair tests useful for probing the pathological mechanism of depression and for the evaluation of antidepressant drugs [39]. These tests are sensitive to all major classes of antidepressant drugs including tricyclics, serotonin reuptake inhibitors, monoamine oxidase inhibitors, and atypical [28]. Characteristic behavior scored in both tests is termed immobility, reflecting behavioral despair as seen in human depression [31]. The results presented here show, to our knowledge for the first time, that UL_EtOH_ given orally is effective in producing significant antidepressant-like activity, when assessed in FST and in TST. The antidepressant-like activity of UL_EtOH_ in FST and TST was not comparable but weaker than that of IMI, used as a standard antidepressant in a dose of 10 mg/kg.

In FST and TST, psychostimulants are also shown to reduce immobility but in contrast to antidepressants they cause a marked motor stimulation. Locomotor activity test was also observed after UL_EtOH_ treatment. We employed an additional locomotor activity test to check the motor stimulating activity of UL_EtOH_ after tests. These results suggested that UL_EtOH_, at the same doses that produce an antidepressant-like activity, did not show significant locomotor stimulation. The antidepressant-like activity of UL_EtOH_ is specific.

The precise mechanisms by which UL_EtOH_ produced antidepressant-like activity are not completely understood. However, according to our results, the antidepressant-like activity of UL_EtOH_ was additive to the treatment of animals with IMI (a-NE/5-HT reuptake inhibitor), FLU (a selective 5-HT reuptake inhibitor), CLO (a selective MAO-A inhibitor), and KET (a preferential 5-HT_2A_ receptor antagonist) when tested in FST and TST. This effect was not accompanied by hyperlocomotion (data not shown) that could produce a false-positive antidepressant-like activity. These suggest that UL_EtOH_ might produce antidepressant-like activity by interaction with monoamines receptors, and monoamine oxidase, thereby increasing the levels NE, 5-HT, and DA in the brains of mice and was related to downregulation of 5-HT_2A_ receptor (inhibition of 5-HT_2A_ receptor expression exerts antidepressant-like activity) [40]. Moreover, this study suggests that the combination of UL_EtOH_ with these antidepressants might be helpful in the treatment of depression.

Intensive research into the neurobiology of depression suggests that an increase in the monoamine levels at the synapse is believed to be the first step in a complex cascade of events that results in antidepressant activity [41]. Four brain regions were studied: the frontal cortex, the striatum, the hippocampus, and the hypothalamus, which are involved Integrating in important behavioral functions, such as emotion, motivation, and learning and memory [41, 42]. Abnormal monoamine levels in four brain regions may be relevant to the depressed state. Our results show that UL_EtOH_ increased the levels of 5-HT and 5-HIAA in cortex, striatum, hippocampus, and hypothalamus, the levels of NE and MHPG in cortex and hippocampus, the level of NE in striatum, and the level of DOPAC in striatum. The HPLC assay showed that a significant increase in DOPAC in the striatum was observed after UL_EtOH_ treatment. The results from behavior and HPLC assay were inconsistent possibly because behavioral changes are not significantly sensitive to small changes in dopamine level in the brain. Integrating the HPLC, and pharmacological treatments results, we inferred that the anti-depression mechanism of UL_EtOH_ might be partly due to its influence on the function of 5-HT,NE systems through the regulation of serotonergic and adrenergic receptors and/or the metabolism of 5-HT and NE.

MAO exists in two subtypes, A and B. The original MAOIs are nonselective, inhibiting both forms. The A form of MAO preferentially metabolizes 5-HT and NE, the monoamines most closely linked to depression. The B form preferentially metabolizes trace amines, including phenethylamine. MAO-A and MAO-B metabolize DA and tyramine [43]. Hou et al. [44] concluded that the (+) catechin and (−) epicatechin of methanol extract of *UR* had inhibitory effect on MAO-B activity. However, due to the different extracting methods in this research, we did not detect the contents of (+) catechin and (−) epicatechin in UL_EtOH._ (unpublished data). Furthermore, based on the same studies have reported a positive correlation between oxidative stress and depression [45], and *Gouteng *has antioxidant activity [46]. We applied two-weeks oral administration of UL_EtOH_ to conduct monoamine oxidase activity test. The results of our study reveal that UL_EtOH_ inhibited MAO-A activity.

Several studies demonstrated that the herb extract and its active component RHY protect neurons against the ischemia, glutamate-, or dopamine-induced damage or death [20, 21, 47], and regulation of monoamine transporters [23, 24]. From the above studies, we inferred that RHY might be the main active component in *Gouteng's* antidepression activity. Further studies are needed to verify the antidepressant activity of RHY and underlying mechanisms.

In conclusion, UL_EtOH_ contained most RHY among *Uncaria* species of *Gouteng* in Taiwan. UL_EtOH_ showed antidepressant-like activity in FST and TST. The mechanism of anti-depressive-like activity of UL_EtOH_ was mediated by increasing the monoamines level, particularly 5-HT and NE in different brain regions of mice. Furthermore, UL_EtOH_ was proofed to inhibit the activity of MAO_A_. From the present study, we conclude that UL_EtOH_ is a worth developing Taiwanese specific medicinal plant, and thus we suggest that it should be included in Pharmacopoeia.

## Figures and Tables

**Table 1:** Effect of UL_EtOH_ on the concentration (ng/g tissue) of monoamines and their metabolites in the cortex of mice brain.

Groups	Cortex (ng/g tissue)					
	NE	MHPG	DA	DOPAC	5-HT	5-HIAA
Normal	525.96 ± 43.71	280.41 ± 12.76	735.26 ± 53.40	625.26 ± 63.78	438.75 ± 36.89	372.32 ± 18.22
Control versus FST	168.49 ± 17.15^###^	119.06 ± 13.32^#^	596.01 ± 27.13	530.69 ± 49.32	216.67 ± 25.11^##^	143.76 ± 31.54^###^
Imipramine 10 mg/kg	583.92 ± 28.9***	233.37 ± 59.00	701.97 ± 56.63	411.47 ± 49.71	711.42 ± 63.27***	342.13 ± 18.26**
UL_EtOH_ 0.125 g/kg	146.39 ± 10.11	176.81 ± 29.29	834.17 ± 55.66	395.71 ± 26.67	346.30 ± 8.91	167.40 ± 31.30
UL_EtOH_ 0.25 g/kg	292.78 ± 26.93*	145.86 ± 18.35	695.69 ± 29.25	433.10 ± 16.43	499.49 ± 19.78***	165.02 ± 46.22
UL_EtOH_ 0.5 g/kg	320.69 ± 20.16**	273.19 ± 58.11*	622.98 ± 67.15	577.92 ± 88.01	639.27 ± 49.86***	311.63 ± 23.23**

Values were the mean ± SEM (*n* = 6). ^#^ *P* < 0.05, ^##^ *P* < 0.01, ^###^ *P* < 0.001 as compared with the normal group. *P < 0.05, **P < 0.01, ***P < 0.001 as compared with the control versus FST group (one-way ANOVA following by Bonferroni's test).

**Table 2:** Effect of UL_EtOH_ on the concentration (ng/g tissue) of monoamines and their metabolites in the striatum of mice brain.

Groups	Striatum (ng/g tissue)					
	NE	MHPG	DA	DOPAC	5-HT	5-HIAA
Normal	408.22 ± 74.25	80.23 ± 84.83	1021.35 ± 74.58	1256.23 ± 160.42	334.60 ± 65.88	242.45 ± 21.90
Control versus FST	331.74 ± 33.07	75.23 ± 45.83	681.62 ± 69.78	927.96 ± 198.73	103.09 ± 44.16^#^	140.02 ± 50.36
Imipramine 10 mg/kg	696.74 ± 83.8**	78.71 ± 17.44	950.93 ± 58.4	1032.17 ± 160.68	426.01 ± 60.75*	352.37 ± 87.32
UL_EtOH_ 0.125 g/kg	466.40 ± 33.77	61.58 ± 7.65	835.30 ± 92.92	1864.85 ± 272.97*	241.71 ± 29.87	254.62 ± 36.12
UL_EtOH_ 0.25 g/kg	467.62 ± 24.12	110.10 ± 38.63	735.40 ± 61.14	1873.61 ± 189.68*	356.64 ± 73.28*	508.37 ± 43.27***
UL_EtOH_ 0.5 g/kg	860.02 ± 67.30***	129.91 ± 16.13	743.81 ± 86.79	1847.19 ± 182.61*	420.20 ± 94.71*	751.38 ± 34.33***

Value were the mean ± SEM (*n* = 6). ^#^ *P* < 0.05 as compared with the normal group. *P < 0.05, **P < 0.01, ***P < 0.001 as compared with the control group (one-way ANOVA following by Bonferroni's test).

**Table 3:** Effect of UL_EtOH_ on the concentration (ng/g tissue) of monoamines and their metabolites in the hippocampus of mice brain.

Groups	Hippocampus (ng/g tissue)					
	NE	MHPG	DA	DOPAC	5-HT	5-HIAA
Normal	581.57 ± 25.89	640.84 ± 44.27	713.98 ± 27.06	639.43 ± 49.15	638.07 ± 31.74	613.67 ± 78.51
Control versus FST	183.67 ± 24.01^#^	210.34 ± 22.72^##^	275.21 ± 34.86^##^	268.46 ± 26.14	113.72 ± 25.71^###^	192.77 ± 15.70^##^
Imipramine 10 mg/kg	851.76 ± 96.22***	682.1 ± 31.39***	633.56 ± 63.63	445.35 ± 94.67	898.71 ± 35.47***	1028.46 ± 75.43***
UL_EtOH_ 0.125 g/kg	431.38 ± 52.01	386.16 ± 27.86	435.43 ± 63.16	360.87 ± 107.15	201.48 ± 73.29	354.73 ± 91.78
UL_EtOH_ 0.25 g/kg	510.95 ± 52.67**	515.38 ± 97.48**	539.59 ± 72.49	432.42 ± 67.79	229.74 ± 55.82	509.97 ± 44.55*
UL_EtOH_ 0.5 g/kg	824.21 ± 71.69***	735.31 ± .84***	612.29 ± 84.59	532.09 ± 42.28	381.89 ± 10.25**	665.73 ± 42.55**

Value were the mean ± SEM (*n* = 6). ^#^ *P* < 0.05, ^##^ *P* < 0.01, ^###^ *P* < 0.001 as compared with the normal group.

*P < 0.05,**P < 0.01,***P < 0.001 as compared with the control group (one-way ANOVA following by Bonferroni's test).

**Table 4:** Effect of UL_EtOH_ on the concentration (ng/g tissue) of monoamines and their metabolites in the hypothalamus of mice brain.

Groups	Hypothalamus (ng/g tissue)					
	NE	MHPG	DA	DOPAC	5-HT	5-HIAA
Normal	162.87 ± 18.84	359.47 ± 42.85	673.24 ± 39.45	632.91 ± 31.21	82.70 ± 1.59	610.04 ± 52.00
Control versus FST	82.12 ± 10.34^#^	301.73 ± 33.83	368.95 ± 24.42^##^	359.99 ± 20.43	27.61 ± 4.24^###^	250.69 ± 51.46^##^
Imipramine 10 mg/kg	491.34 ± 17.78**	750.59 ± 67.95**	466.00 ± 49.59	498.45 ± 12.95	72.86 ± 5.06***	780.53 ± 78.00***
UL_EtOH_ 0.125 g/kg	86.59 ± 20.24	256.23 ± 28.26	311.62 ± 49.34	344.75 ± 46.98	37.00 ± 6.16	612.67 ± 65.72**
UL_EtOH_ 0.25 g/kg	161.51 ± 11.41	357.50 ± 22.94	407.61 ± 23.02	564.04 ± 42.35	40.99 ± 3.46	633.35 ± 65.61**
UL_EtOH_ 0. 5 g/kg	148.62 ± 13.21	501.39 ± 57.27	439.88 ± 51.01	585.68 ± 26.87	86.84 ± 11.23***	877.39 ± 75.70***

Value were the means ± SEM (*n* = 6). ^#^ *P* < 0.05, ^##^ *P* < 0.01,  ^###^ *P* < 0.001 as compared with the Normal group.

**P < 0.01,  ***P < 0.001 as compared with the control group (one-way ANOVA following by Bonferroni's test).

**Table 5:** Effects of UL_EtOH_ (0.125, 0.25, 0.5 g/kg, p.o.) and clorgyline (10 mg/kg, i.p.) for two weeks' administration on MAO-A, MAO-B activity in mouse brain.

Group	MAO-A activity (% of mouse brain)	MAO-B activity (% of mouse brain)
Control	98.56 ± 4.44	96.89 ± 4.10
Clorgyline 10 mg/kg	45.05 ± 8.02**	102.41 ± 5.50
UL_EtOH_ 0.125 g/kg	107.31 ± 14.21	103.86 ± 7.46
UL_EtOH_ 0.25 g/kg	80.97 ± 7.20	107.70 ± 5.50
UL_EtOH_ 0.5 g/kg	49.84 ± 7.02**	105.94 ± 3.99

Value were the mean ± SEM (*n* = 6). **P < 0.01 as compared with the control group (one-way ANOVA following by Bonferroni's test).

## Abbreviations

5-HIAA: 5-hydroxyindoleacetic acid
5-HT: Serotonin
BUP: Bupropion
CLO: Clorgyline
DA: Dopamine
DOPAC: 4-dihydroxyphenylacetic acid
FLU: Fluoxetine
FST: Forced swimming test
HPLC: High-performance liquid chromatograph
HRP: Horseradish peroxidase
IMI: Imipramine
KET: Ketanserin
MAOIs: Monoamine oxidase inhibitors
MAP: Maprotiline
MHPG: 4-Hydroxy-3-methoxyphenylglycol
NE: Norepinephrine
PBS: Phosphate-buffered saline
RHY: Rhynchophylline
SNRIs: Specific serotonin-norepinephrine reuptake inhibitors
SSRIs: Selective serotonin reuptake inhibitors
TCAs: Tricyclic antidepressants
TST: Tail suspension test
UH: *Uncaria hirsuta* Haviland
UL: *Uncaria lanosa* Wallich. var*. appendiculata* Ridsd
UR: *Uncaria rhynchophylla* (Miquel) Jacks.
